# Validating the Delone and Mclean’s model in a developing country’s infectious disease pandemic context.

**DOI:** 10.1186/s12879-024-09483-x

**Published:** 2024-06-17

**Authors:** Uche Ikenyei, Nicole Haggerty

**Affiliations:** 1 Western University, London, ON Canada; 2 Ivey Business School, Western University, London, ON Canada

**Keywords:** Health Information Systems (HIS), Infectious disease pandemic readiness and response, DeLone & McLean’s Information systems Success model, Developing countries

## Abstract

**Background:**

This study aimed at validating the updated DeLone and McLean’s information systems success model (D&MISS) in a developing country’s infectious disease pandemic preparedness and response context. The findings from this study are relevant to inform policies and actions for enhancing developing countries’ the Health Information System’s (HIS) performance, and specifically to improve their future pandemic readiness and response. The study sought to respond to a key research question: to what extent can the D&MISS model provide evidence to enhance the HIS’s infectious disease pandemic readiness and response in developing countries?

**Method:**

A cross-sectional study design that involved a multi-stage probability sampling approach to select eligible healthcare workers was applied. Conducted in Nigeria and Liberia, 576 primary healthcare workers, out of the proposed 600, participated, representing a response rate of 96%. The D&MISS model served as the theoretical underpinning for this study, and nine hypothesized relationships were stated before the study based on the interconnectedness of the model’s six dimensions. Structural Equation Modelling (SEM) data analysis using the Partial Least Square approach was used to determine if hypothesized relationships were supported.

**Results:**

70% of the observed variance in the Net Benefit construct was explained by the predictive influence of the Use and User Satisfaction constructs. The Use construct had a slightly more substantial predictive influence than the User Satisfaction construct. Eight of the nine hypothesized relationships were supported, except for the relationship between Information Quality and Use. The relationships between System Quality and Use and User Satisfaction and Net Benefit had the highest beta coefficient, statistically significant at *p* < 0.05.

**Conclusion and relevance:**

The D&MISS model demonstrated its relevance in providing evidence on the gaps of the HISs regarding future pandemic preparedness and response. However, from a future research opportunity, its enhancement and modifications with context-specific dimensions peculiar to developing countries will improve its ability to provide more context-specific evidence to improve pandemic preparedness and response for developing countries.

**Supplementary Information:**

The online version contains supplementary material available at 10.1186/s12879-024-09483-x.

## Background

Till date, the occurrences of infectious disease pandemics have continued to rise globally [1, 2]. Infectious disease pandemics have become more frequent and unpredictable, with devastating impacts withnessed through high morbidity and mortality rates during and after pandemic events. This challenge cuts across geographical locations, especially in developing countries where health systems and health information systems (HIS) capacity for pandemic preparedness and response is weak, making these regions more vulnerable to epidemic shocks [3].

From the clinical context, we define the HIS as the information-generating block of the health systems, in either traditional paper-based format or technology-based platforms; during routine clinical visits or public health emergencies such as infectious disease outbreaks or pandemics, it collects and collates patient-level health information; analyzes this collection of patient information to generate evidence, and presents it in visually acceptable formats to empower decision-makers with the information they need to act promptly within and beyond clinical settings and to formulate essential lifesaving policies that will ultimately improve population health outcomes [4, 5].

Poor-performing and ill-prepared HISs, especially in developing countries, have far-reaching consequences on population health outcomes during pandemic events. For instance, during the 2013–2016 Ebola Virus Disease (EVD) pandemic, 28,652 cases and 11,325 deaths were recorded in Guinea, Sierra Leone, and Liberia, accounting for over 99% of the global cases [6]. Had the shortcomings of the HISs of these three countries been identified and addressed on time, their HISs would have been more responsive to provide the necessary evidence for actions and policies before and during the pandemic to prevent or significantly reduce the impact of the pandemic [7, 8].

One way of identifying HIS gaps is through HIS research. Theoretical underpinned HIS research have demonstrated immense value in enabling stakeholders to identify their pandemic readiness gaps. Among several, the DeLone and McLean Information Systems Success Model (D&MISS) stands out as a viable information system (IS) model that has demonstrated its widespread applicability and significance in numerous IS contexts [9, 10]. However, despite the D&MISS model’s demonstrated value in various IS studies, evidence gaps remain in the model’s utility specific to public health emergencies such as infectious disease pandemics. Furthermore, ample evidence exists on the model’s relevance in electronic-based health information systems in developed countries. Yet, limited knowledge exists of the model’s validation and applicability in emergency contexts such as infectious disease pandemic preparedness and response in primary healthcare clinics and developing countries where paper-based HIS persists.

Applying the D&MISS model as a theoretical underpinning for developing countries will enable these countries to make significant pandemic preparedness and response preprations and progress in future pandemics. However, more importantly, having a better understanding of how the different IS success factors helps to better understand the pandemic readiness and performance of the paper-based HIS at the primary healthcare (PHC) level in several developing countries has become paramount and urgent. This is particularly very important considering that the more accessible PHC is the primary point of care at the community level where evidence is frequently generated to better understand the pandemic in several developing countries [11]. It is important to recognize the potential for future pandemics to have similarly or more devastating effects if the HIS gaps at the PHC level are left unaddressed before the next pandemic occurs.

Therefore, the primary objective of this study was to validate the D&MISS model within the primary healthcare paper-based HIS setting in two developing countries and within an infectious disease pandemic preparedness and response context. Validating the model in these contexts will provide the critical evidence needed to demonstrate that the model is a viable model that can support HIS pandemic readiness research in developing countries, particularly in countries where paper-based HIS persists. The results of this study will also provide insights for informed policymaking and actions aimed at enhancing the performance of PHC-level HIS in several developing countries. This will aid in developing a more resilient HIS equipped to provide robust evidence support during future infectious disease pandemics.

## Methods

The research in this paper builds upon the updated DeLone and McLean model as its theoretical foundation and adapts definitions from other D&MISS published papers in the literature in defining its dimensions. While the updated DeLone and McLean model’s analytical approach was designed to be causal or temporal, Iivari’s study projected the model’s predictive capabilities [12]. In this study, the predictive capability was exploited to identify how different IS factors predicted the success of the HIS in PHC settings for future infectious disease pandemic preparedness and response in developing countries. This predictive approach retains the model’s six interrelated constructs but shifts the focus from causal or temporal relationships to exploring predictive interrelationships.

By adopting the predictive capabilities of the D&MISS model, the study aimed to determine the most influential factor predicting and hindering the performance of the HIS in developing countries’ context as a means of improving their pandemic preparedness and response. This study identifies six constructs and 24-item measures carefully selected to ensure comparability with other D&MISS studies, as suggested by DeLone and McLean. Table 1 lists the item measures in each construct, their construct validity and internal consistency, and each construct was defined as follows.

**Table 1:** Construct Validity and Internal Consistency

Constructs & Item measure	Outer Loading	CR	AVE
System Quality		**0.89**	**0.55**
SQY 1: Ease of use (EOU)	0.87		
SYQ 2: Usefulness	0.92		
SYQ 3: Reliability	0.93		
SYQ 4: Availability	0.91		
SYQ 5: Perceived importance of health information technology (HIT)	**0.40**		
SYQ 6: Availability of health information technology (HIT)	**0.45**		
SYQ 7: Availability of government-approved patient records tools	**0.45**		
Information Quality		**0.95**	**0.80**
IQY 1: Timeliness	0.90		
IQY 2: Accuracy	0.90		
IQY 3: Completeness	0.90		
IQY 4: Culture of information use	0.87		
IQY 5: Promotion of information use	0.88		
Service Quality		**0.89**	**0.55**
SEQ 1: The satisfaction of the user guide	0.86		
SEQ 2: Adequate technical support	0.89		
SEQ 3: Knowledgeable	0.84		
Use		**0.89**	**0.67**
U 1: Use of HIS	0.82		
U 2: Frequency of use	0.78		
U 3: Perceived usefulness	0.84		
U 4: Dependency on the HIS	0.84		
User Satisfaction		**0.96**	**0.92**
US 1: Overall user satisfaction	0.96		
US 2: Improved dependence	0.96		
Net Benefits		**0.96**	**0.89**
NB 1: Improved decision making	0.94		
NB 2: Improved effectiveness	0.93		
NB 3: Effective communication	0.95		

*System Quality (SYQ)*: the HIS’s ability to receive, process, and make patient-specific information accessible for decision-making. Seven item measures were applied to assess system quality.

*Service Quality (SEQ)*: the extent of HIS service support provided to the system’s teams. Three item measures were applied to assess service quality.

*Information Quality (IQ)*: the excellence and dependency of the final product derived from the HIS after-data analysis. Five item measures were used to measure information quality.

*User Satisfaction (US)*: the level of contentment experienced while using the HIS to perform tasks. Two-item measures were used to evaluate user satisfaction.

*Use (U)*: the application of the HIS in making evidence-based decisions. Three item measures were used to assess use.

*Net Benefits (NB)*: This study adopts an individual level of analysis, as suggested by the updated DeLone and McLean’s model. Net benefits are the results, products, and advantages of using the HIS. Three item measures were used to measure net benefits.

## Hypothesis

**Fig. 1:**
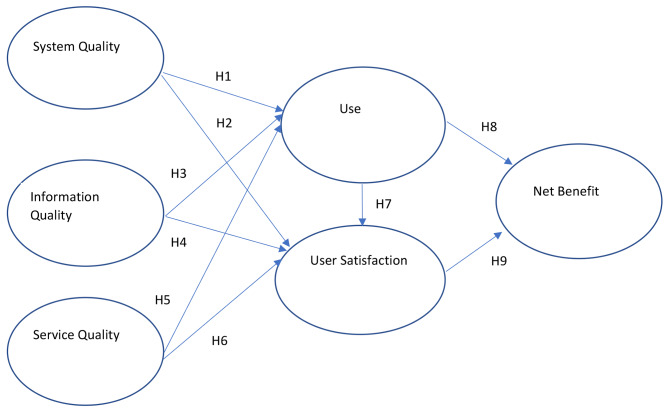
Research model

The study puts forth nine hypothetical relationships, each aiming to showcase a positive predictive interdependence relationship between the six constructs. Figure 1 presents the research model of these pathways, which include the following relationships:

H1: System quality positively predicts use.

H2: System quality positively predicts user satisfaction.

H3: Information quality positively predicts use.

H4: Information quality positively predicts user satisfaction.

H5: Service quality positively predicts use.

H6: Service quality positively predicts user satisfaction.

H7: Use will positively predict user satisfaction.

H8: Use will positively predict perceived net benefit.

H9: User satisfaction positively predicts net benefit.

### Research context

This study took place in Liberia and Nigeria. Each country was selected due to their distinct lived and past experiences with the 2013–2016 EVD and the 2019 Corona Virus Disease (COVID) -19 pandemics, respectively. Liberia’s high EVD death rate and Nigeria’s significant COVID-19 prevalence in Africa made them appropriate countries of choice. Data collection occurred between January and April 2021, as soon as ethical approval in each jurisdiction was received.

### Study design and validity of the data collection tools

A survey questionnaire facilitated data collection for this study’s observational, cross-sectional research design. To ensure measurement accuracy, the questionnaire underwent face and content validity validation. For face validity, selected participants rated the 24-item measures on a 7-point Likert scale. Four Ph.D. students, two from Nursing and two from Health Information Sciences at The University of Western Ontario, Canada evaluated the study’s instrument for face validity. With backgrounds as Nurses or Health Information Management specialists in African countries, they reviewed each item measure to ensure alignment with the intended dimensions and audience.

After completing face validity, seven health care and health system experts, including University of Western Ontario-trained graduate students and professionals from African countries, were engaged to ensure the content validity of the data collection tools. These experts reviewed and refined the draft instrument’s item measures, providing a content validity index (CVI) rating for each. Their feedback led to the modification of the survey tool’s content. Items were adjusted or removed based on the experts’ rankings and suggestions, ensuring both face and content validity for the data collection tool.

### Sample size

The respondents were primary healthcare workers from randomly selected clinics who were responsible for, in one way or another, managing the HIS during their pandemic events. A sample size of 600 PHC workers, 300 per country, randomly selected from 200 PHCs, 100 per country, was proposed. The proposed sample size was based on past studies that demonstrated the threshold sample size needed for Structural Equation Modelling (SEM) and this study ensured that a higher than the recommended range of 100–150 participants required for conducting SEM was achieved [13–15].

### Sampling

This study employed a multi-stage probability sampling approach to ensure external validity. This involved using a combination of sampling methods applied at various stages, starting from country-wide data on health facilities to ultimately selecting the eligible respondents from selected PHCs. A four-stage multi-stage sampling was used in Liberia consisting of four stages, each building upon the previous one. The first stage utilized a stratified random sampling method, unique for its approach in identifying high and low EVD burden. In the second stage, a simple random sampling method was applied using Microsoft Excel’s function to select two eligible counties one each from an EVD high and low burden region, ensuring representation from each homogeneous category. Stage three employed a probability proportionate to size sampling method, ensuring proportional representation across selected counties. Finally, in stage four, another simple random sampling method was used to select primary health centres (PHCs) from the list, guided by Excel’s random selection. This process facilitated the final selection of eligible healthcare workers from 100 randomly chosen primary health centres (PHCs) out of Liberia’s 774 available at the time of the study.

Similarly, in Nigeria, with over 33,000 PHCs, a three-stage multi-stage probability sampling with a combination of the sequential steps of stratified, probability proportion to size, and simple random sampling was used to propose 300 healthcare workers from 100 PHCs. The multi-stage probability sampling method was selected to ensure each respondent from a country-level study had an equal and unbiased chance of being selected for the study.

### Data collection

Data collection occurred from January to April 2021 amidst significant international travel limitations due to global COVID-19 transmission. Two lead consultants, each highly experienced in monitoring and evaluating health programs, were hired—one for each country—to supervise data collection, overseen by the primary author UI. Additionally, ten resident data collectors were employed in each country to collect data from randomly selected primary healthcare clinics (PHCs) in the four selected states. The primary author, UI conducted four virtual training sessions via Zoom to train the two lead consultants on data collection tools and logistics. In person, the lead consultants trained the data collectors and printed the paper questionnaires in Nigeria and tablets in Liberia. Paper questionnaires were used in Nigeria, while tablets downloaded with Kobo Toolbox with the questionnaires already designed into the app were utilized in Liberia for data collection and transmission. In Nigeria, data was entered into the Kobo Toolbox daily and reviewed by the primary author UI and lead consultant for quality control. Subsequently, cleaned data was exported to Smart PLS version 3.3.3 for Structural Equation Modelling (SEM) analysis.

### Data analysis

Smart PLS version 3.3.3 13 was the data analytical tool for conducting Structural Equation Modelling (SEM) analysis. Amongst other data derived from SEM, the analysis focused on determining path coefficients and *P*-values to determine supported relationships between constructs. R^2^ was used to explain the predictive influence of the exogenous variables on the endogenous variables. Discriminant and construct validity and internal consistency were also derived using the Smart PLS software.

### Ethical approval

Western University’s ethical board provided ethical approval in August 2020, the University of Liberia’s Pacific Institute for Research and Evaluation Institution Review Board (UL-PIRE-IRB) in January 202, the National Health Research Ethics Committee of Nigeria (NHREC) in February 2021 and the Lagos State Primary Health Care Board in April 2021.

### Consent to participate

All questionnaires included an informed consent section where respondents indicated their willingness to participate or decline. Interviews were conducted only with the consent of the respondents. To ensure confidentiality, the questionnaire deliberately omitted questions that could reveal personal identification, such as names and job titles.

## Results

### Descriptive statistics

The participation response rate was 101% in Nigeria, with 302 healthcare workers participating, surpassing the intended proposed sample size of 300. Two additional participants volunteered to participate after observing their colleagues completing the questionnaires in Lagos State. In Liberia, 276 individuals participated, representing 92% of the intended 300 healthcare workers. The combined response rate for the two countries was 96%.

### Structural Equation Modelling (SEM) statistics

SEM analysis was performed on the combined data from Nigeria and Liberia. The Partial Least Square Structural Equation Modelling (PLS-SEM) method, instead of the covariance-based SEM commonly used in causal or temporal research, was adopted for data analysis. PLS-SEM was chosen due to its predictive analysis capability.

### Model fit

It is recommended that R^2^ and factor analysis be presented for model fit, as they provide more informative data about the model’s fit for predictive SEM analysis than traditional goodness-of-fit index measures typically found in causal-based SEM analysis [16].

### R^2^ and factor loading

Following recommendations by past authors, a cut-off value of 0.7 was adopted to determine the outer loading of the item measures [16]. Item measures with an outer loading below 0.7 were excluded from the measurement model to ensure the construct’s reliability. As a result, SYQ5, SYQ6, and SYQ7 were removed from the system quality construct before proceeding with the SEM analysis. Table 1 presents the outer loading values for the 24 item measures selected for the SEM analysis, including the three item measures that failed to meet the criteria for further analysis.

**Fig. 2:**
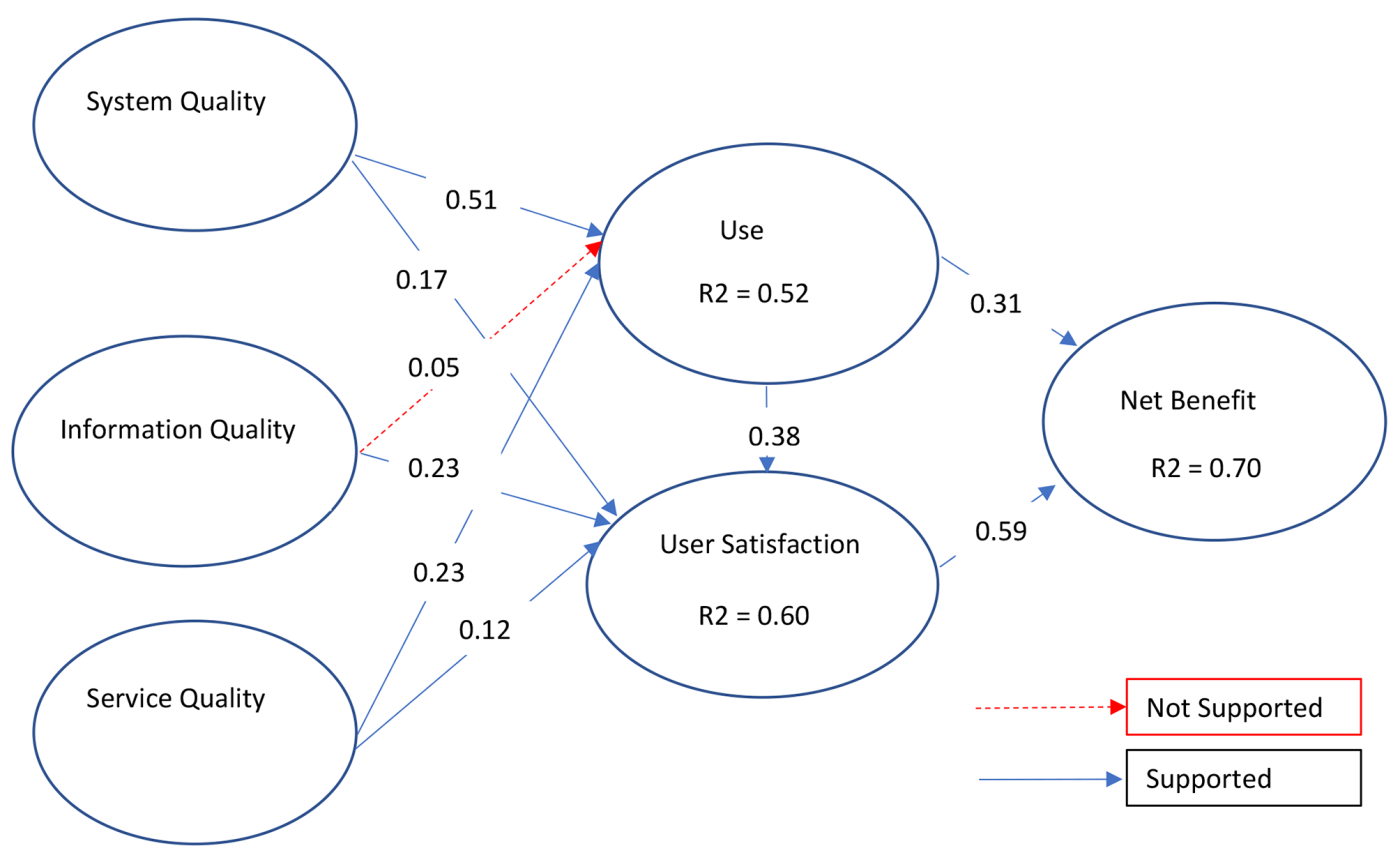
Structural Model demonstrating R^2^ and Factor Loading

Figure 2 presents the R^2^ values for the Use, User Satisfaction, and Net Benefit constructs. The R^2^ value represents the variance attributed to the effects of the exogenous variables on the endogenous variables. In this study, the Use and User Satisfaction constructs were exogenous variables for the Net Benefit construct. This indicates that 70% of the variance observed in the Net Benefit construct was attributed to the predictive influence of the Use and User Satisfaction constructs, with the Use construct exhibiting a slightly more substantial predictive influence than the User Satisfaction construct.

Moreover, the combined effects of the three quality constructs, namely System Quality, Information Quality, and Service Quality, accounted for 52% and 60% of the variance found in the Use and User Satisfaction constructs, respectively.

### Construct validity

This study established convergent validity using the average variance extracted (AVE), while discriminant validity was assessed by verifying if the square root of the latent construct’s AVE was higher than the correlation of the constructs in the matrix [17]. AVE of ≥ 0.5 cut-offs has been deemed ideal for construct item measures [18]. The AVE for each construct is also presented in Table 1, while Table 2 shows that the model has satisfactory divergent validity.

**Table 2:** Discriminant validity

Constructs	IQ	NB	SEQ	SYQ	U	US
IQ	**0.89**					
NB	0.74	**0.94**				
SEQ	0.62	0.67	**0.86**			
SYQ	0.79	0.80	0.66	**0.93**		
U	0.58	0.73	0.61	0.67	**0.82**	
US	0.65	0.81	0.61	0.69	0.70	**0.96**

### Internal consistency

Internal consistency is a key measure to determine a model’s reliability. A model is considered reliable if its Composite Reliability (CR) score equals or exceeds 0.7 [19, 20]. Table 1 also presents the CR scores for each construct in the model, indicating that all constructs had a CR of 0.7 or higher, demonstrating the measurement model’s internal consistency.

Table 3 presents data on regression path coefficients and *p*-values. The regression path coefficients indicate the presence of relationships between the hypothesized constructs, while the *p*-values determine if these relationships are statistically significant or occurred by chance. According to Table 3, the three quality constructs had a positive predictive relationship with the Use and User Satisfaction constructs except for one relationship. System quality positively predicted both Use (β = 0.51, *p* < 0.05) and User Satisfaction (β = 0.17, *p* < 0.05). Information Quality positively predicted User Satisfaction (β = 0.23, *p* < 0.05) but not Use (β = 0.05, *p* > 0.05). Service Quality positively predicted both Use (β = 0.23, *p* < 0.05) and User Satisfaction (β = 0.12, *p* < 0.05).

**Table 3:** Paths Coefficients

	Path	β	*P*-Value	Conclusion
H1	System quality → use	0.51	0.00	Supported
H2	System quality → user satisfaction.	0.17	0.00	Supported
H3	Information quality → Use	0.05	0.26	Not Supported
H4	Information quality → User satisfaction	0.23	0.00	Supported
H5	Service quality → Use	0.23	0.00	Supported
H6	Service quality → User satisfaction	0.12	0.01	Supported
H7	Use → user satisfaction	0.38	0.00	Supported
H8	Use → Net benefits	0.31	0.00	Supported
H9	User satisfaction → Net benefit	0.59	0.00	Supported

*In a one-tailed test, the level of significance was determined at a p-value < 0.05*.

### Hypothesis testing

The results support the hypothesized positive predictive relationships between the three quality constructs and Use and User Satisfaction, except for the relationship between Information Quality and Use, for which there was insufficient evidence to conclude a predictive relationship. On the other hand, the relationship between User Satisfaction and Net Benefits had the highest β coefficient of 0.59, indicating a positive predictive relationship that was statistically significant at *p* < 0.05.

## Discussion

This section based on the results from the analysis aims to proffer policy-relevant insights that shed light on significant implications for improving pandemic preparedness and response in developing countries, particularly countries where paper-based HIS still prevails in their PHCs. The insights gained from the results can provide valuable guidance for policymakers, healthcare decision-makers, and individuals responsible for managing a pandemic response in a developing country setting.

First and foremost, in all the supported relationships, decision-makers should endeavour to increase budgetary allocation to improve the ease of healthcare workers utilizing their HIS during pandemic events [8]. Additionally, streamlining the process of recording patient data, perhaps by reducing their reliance on paper records, is increasingly becoming important. Such streamlining should aim to make data documentation more efficient and less burdensome for healthcare workers, who already face immense pressure during pandemics to provide care and simultaneously document services provided to patients simultaneously. By simplifying these tasks through digital platforms, healthcare workers can devote more time to critical patient care while making data entry seamless, steps that will significantly improve their overall pandemic response. Additionally, a simple approach, such as creating dedicated time slots during planning and strategy meetings to pull up and discuss the routine or public health emergency data from a lens of improving their pandemic readiness, is not only cost-efficient but also very practical and productive at the community level.

Decision-makers should increase funding to ensure that healthcare workers managing the HIS receive routine and ad hoc support from oversight officers from the local government areas where the healthcare facilities are located. The technical support provided to healthcare workers should not be limited to improving the capacity of healthcare workers to manage their HIS and the satisfaction that comes from utilizing their HIS but also to supporting decision-makers during routine healthcare services and pandemic events. This investment to improve Service Quality will significantly improve Use and User Satisfaction and enhance the HIS’s capacity to support decision-makers during pandemics.

One intriguing finding was the unsupported relationship between Information Quality and Use. It is feasible that healthcare workers may not fully appreciate the value of Information Quality in influencing HIS Use during pandemics. Decision-makers should invest in activities aimed at changing this perception. One way of achieving this is by increasing routine data quality assessments (DQA). DQAs reveal data quality gaps from the HISs, and these gaps, once identified, can prompt healthcare workers to routinely utilize their HISs more effectively to rountinely produce good quality data for rountine and periodic decision before a pandemic event.

Lastly, this study underscores the importance of User Satisfaction in predicting Net Benefits. Decision-makers should consider conducting routine simulation exercises demonstrating how accurately HIS Use and User Satisfaction can enhance decision-making quality during pandemics. This is a compelling incentive for healthcare workers to embrace their HIS, recognize its value, and improve patient care during pandemics.

### Implication of the findings on the theoretical approaches in HIS research

Despite proving the DeLone and McLean model’s utility in this HIS research, it was evident that the current six constructs of the D&MISS model is not sufficient to provide nuanced evidence on predictive factors for the three quality dimensions (Information, System and Service Quality) in a way that makes the model more responsive to providing holistic and context-specific gaps assessments within the HIS in several developing countries. This limitation impedes the decision-maker’s ability to pinpoint and address context-specific shortcomings within their HIS, particularly in infectious disease preparedness and response. While efforts have been made to auguement the D&MISS model with context-specific dimensions [10], the infectious disease pandemic lens remains unexplored.

## Conclusions

In conclusion, the study’s findings provide valuable guidance for strengthening pandemic preparedness and response in developing countries by improving the performance of health information systems (HIS). By investing in streamlining processes, enhancing data quality, providing consistent technical support, changing perceptions, and promoting user satisfaction, decision-makers can significantly improve the effectiveness of their HIS in-healthcare settings before, during and after pandemic events. These measures will ultimately save lives and improve overall public health confidence and outcomes.

Additionally, while DeLone and McLean’s Information Systems Success (D&MISS) model continues to remain valuable in Information Systems (IS) and HIS research in particular, its enhancement with more dimensions to better understand factors that predict the three quality dimensions will enable more precise resource allocation for HIS pandemic preparedness and response, particularly in Low-Middle-Income-Countries (LMIC)s. This evolution will empower decision-makers with accurate context-specific evidence to make better-informed choices that will improve their HIS effectiveness before, during, and after pandemic events.

### Electronic supplementary material

Below is the link to the electronic supplementary material.

Supplementary Material 1

## Data Availability

This published article and its supplementary information files include all data generated during this study has been made available.
